# Increased Physical Activity at School Benefits Arterial Blood Pressure in Children—A Prospective Follow-Up Cohort Study

**DOI:** 10.3390/ijerph19084662

**Published:** 2022-04-12

**Authors:** Wojciech Kolanowski, Katarzyna Ługowska, Joanna Trafialek

**Affiliations:** 1 Faculty of Health Sciences, Medical University of Lublin, 20-400 Lublin, Poland; 2 Faculty of Medical and Health Sciences, Siedlce University, 08-110 Siedlce, Poland; katarzyna.lugowska.zdoz@uph.edu.pl; 3 Institute of Human Nutrition Sciences, Warsaw University of Life Sciences, 02-787 Warsaw, Poland; joanna_trafialek@sggw.edu.pl

**Keywords:** children, blood pressure, hypertension, physical activity, prehypertension

## Abstract

(1) Background: A sedentary lifestyle and low physical activity (PA) increase the risk of hypertension in children. The aim of this study was to assess the impact of increased PA at school by elevation of the number of compulsory physical education (PE) lessons on arterial blood pressure in children during a two-year follow-up. (2) Methods: Children (*n* = 245) born in 2007 attending a standard or elevated number of PE lessons in the school timetable (4 and 10 h a week, respectively) took part in the study. Blood pressure was measured starting from age approx. 10 to 12. (3) Results: Starting from a similar level, after 2 years, the percentage of children with normal blood pressure decreased in the standard-PE children from 83.25% to 78.03% but increased in the elevated-PE ones from 83.15% to 86.13%. The prevalence of both prehypertension and hypertension increased by one-third in the standard-PE children from 16.74% to 21.97% but decreased by one-sixth in the elevated-PE ones from 16.85% to 13.87%. The prevalence of hypertension itself increased by one-third in the standard-PE children from 9.82% to 13.12% but decreased in the elevated-PE ones by one-fifth from 9.60% to 7.75% (4) Conclusions: An increase in PA at school by the elevation of the number of PE lessons benefits children’s arterial blood pressure. Early prevention of hypertension in children can be supported by an adequate number of PE lessons in the school timetable.

## 1. Introduction

In recent decades, children have become increasingly sedentary with a low level of physical activity, which elevates the risk of many diseases, including hypertension [1,2]. Moreover, the COVID-19 pandemic, frequent lockdowns, and online learning contributed to a further reduction in the physical activity (PA) of children. Hypertension is one of the most common lifestyle diseases among adults, which usually starts to develop at school age. Hypertension is a life-threatening disease often called a “silent killer” and is a risk factor of cardiovascular diseases and stroke in adulthood. The risk of hypertension is increased by a sedentary lifestyle, excessive body weight, and a high level of salt and saturated fats in the diet [3,4,5]. It is evidenced that hypertension affects younger and younger age groups [5,6,7]. Hypertension currently affects more than 10% of school-age children. Similarly to adults, it more often concerns systolic blood pressure [8,9,10,11]. In Poland, hypertension affects around 12%, and prehypertension 8%, of school-age children [12]. Moreover, the risk of hypertension is 4 to 10 times higher in obese children than in those of a healthy weight [13,14,15].

Elevated blood pressure in children is a growing health problem that is often overlooked [12,13,14,15]. Normal blood pressure figures (systolic and diastolic) in children are calculated based on age, sex, and height, and are available in standardized population percentile grids. Prehypertension is defined as blood pressure in at least the 90th percentile but less than the 95th percentile. Hypertension is defined as blood pressure in the 95th percentile or greater [1,3,13,16,17,18]. It is recommended that blood pressure in children should be measured at least once a year [19,20,21]. Blood pressure increases slightly throughout life as a result of progressive changes in the elasticity of the arteries. It is assumed that in normally developing school-age children, systolic blood pressure increases annually by 1–1.5 mmHg and diastolic by around 0.7 mmHg [22,23]. According to the reference values of percentile grids in Poland, the systolic blood pressure in girls should not exceed 118 mmHg at the age of 10, 120 mmHg at 11, and 122 mmHg at 12; and 117 mmHg, 118 mmHg, and 120 mmHg in 11-, 12-, and 13-year-old boys, respectively [16,17]. In turn, diastolic blood pressure in girls should not exceed 71 mmHg at the age of 10, 72 mmHg at 11, and 73 mmHg at 12; and 70 mmHg, 71 mmHg, and 71 mmHg in 11-, 12-, and 13-year-old boys, respectively [16,17].

It has been shown that in children, PA improves physical fitness, cardiometabolic health, glucose and insulin resistance, and bone health; reduces adiposity; and brings many other health benefits [18,19,20,21,22,23]. Numerous studies have also shown a favourable effect of PA on maintaining normal blood pressure in children [24,25,26,27,28,29,30]. Regardless of the health benefits, many children are reluctant to engage in regular extracurricular PA [24,26,30,31]. Nevertheless, the average level of children’s PA can be increased by elevation of the number of compulsory physical education (PE) lessons in the school timetable. PE is an important element promoting regular PA and an active, healthy lifestyle [31,32,33]. PE lessons at school consist of a diverse range of organized sports activities, such as various aerobic exercises, gymnastics, team games, and athletic forms of movement. The curricular number of PE lessons at primary school in Poland is 4 h a week. However, the generally decreasing level of children’s PA, a predominantly sedentary lifestyle, and increasing risk of related health problems (e.g., hypertension) indicate that in order to improve the health of the younger generation, effective remedial actions are needed to increase the PA level in the entire school-age child population. It was hypothesised that an increase in organized PA at school by an increase in the number of PE lessons in the school timetable might bring many health benefits, including in terms of blood pressure normalization. Consequently, the aim of this study was to assess the impact of increased PA at school by an increase in the number of compulsory PE lessons on systolic and diastolic arterial blood pressure in children during two-year follow-up.

## 2. Materials and Methods

### 2.1. Participants

The study was conducted in six primary schools in Siedlce (a typical medium-sized city in central Poland). The schools had established parallel classes with a standard or elevated number of PE lessons (4 and 10 h a week, respectively), so-called general education classes and sport classes (GC and SC, respectively). Education with an elevated PA at school (SC) began in the 4th grade of school (age approx. 10 years). PE lessons consisted of organised PA in the form of fitness exercises, athletic forms of movement, and team sports games.

Children born in the year 2007, starting the 4th grade of school in the year 2017, took part in the study. The children and their parents declared their informed consent to participate in the study. All of them were informed of the objective of the study and the confidential nature of the results. The approval of the Ethics Committee at Siedlce University (no. 2/2016) was obtained for the study.

A total of 304 children, of which 161 (girls 75; boys 86) attended GC and 143 (girls 67; boys 76) attended SC, were examined. During the study, around 23% of the GC children (*n* = 38) and around 15% of the SC ones (*n* = 21) were excluded from the final analysis. The criteria for exclusion were as follows: absence during one of the measurement sessions, failure to pass to the next grade of school, inappropriate age of the child (other than approx. 10 at the beginning of the study), lack of the child’s consent on the measurement, or diagnosed chronic diseases that may affect the measurement result. However, none of the children’s parents reported problems with diagnosed chronic diseases. The studied classes did not belong to the integration units in which there could be children with health disorders that had an impact on the final results. In cases where a child was admitted to the GC or SC during the study as a result of a transfer from another class or from another school, these results were excluded from the final analysis.

In total, 245 children took part in all measurement sessions (48% girls; 52% boys). The elevated PE group (SC) consisted of 122 children (girls *n* = 61; boys *n* = 61), and the standard PE group (GC, control) consisted of 123 children (girls *n* = 57; boys *n* = 66). The average child’s age at the beginning of the study was 10.27, and at the end, 12.26 years. The final analysis of the results concerned the same group of children who participated in all measurement sessions during the study period.

### 2.2. Procedure

The research was a prospective follow-up cohort study with a control group. It concerned the measurement of systolic and diastolic arterial blood pressure over a period of 2 years in two cohorts of children differing in the number of compulsory PE lessons in the school timetable. The test group included only children with an elevated number of PE lessons (SC, 10 h a week); the control group included only children with a standard number of PE lessons (GC, 4 h a week). The intervention programme was carried out continuously only during school time; during school breaks, the intervention was not carried out. The study consisted of periodic measurements of systolic and diastolic blood pressure in the GC children and the SC ones, starting from the 4th grade of school (age approx. 10 years) when the elevated number of PE lessons was introduced in the SC timetable until the beginning of the 6th grade of school (age approx. 12 years). The measured values were interpreted with reference to the blood pressure percentile grids. Based on this, the percentage of children with normal systolic and diastolic blood pressure, pre-hypertension, and hypertension were determined.

The study was conducted from September 2017 until October 2019, i.e., from age approx. 10 years (4th grade of school) until age approx. 12 years (beginning of the 6th grade of school). The interpretation of the results focused on changes in the systolic and diastolic blood pressure in relation to PA time at school corresponding to the number of PE lessons (GC, SC). The measurements were conducted in schools by members of the research team. The first measurement session (initial) was completed between 1 and 31 September 2017, the second between 1 and 30 March 2018, the third between 1 and 31 September 2018, the fourth between 1 and 30 March 2019, and the fifth (final) between 1 and 31 September 2019. Each child was assigned an identification number and the results were systematically collected and analysed in Microsoft Excel 2016 (Microsoft, Intentional Software, Washington, DC, USA).

### 2.3. Measurements

Systolic and diastolic arterial blood pressure were measured using digital OMRON M3 automatic blood pressure monitors (OMRON Healthcare Europe B.V., Hoofddorp, The Netherlands). The device fulfils the provisions of EC directive 93/42/EEC (Medical Device Directive). Measurements were taken according to the AAMI (Association for the Advancement of Medical Instrumentation) recommendation for electronic devices [34]. Blood pressure measurements were performed in accordance with the recommendations for children [16,17,18]. Before starting the tests, the purpose and method of the measurement were explained to the children. Blood pressure was measured on the right arm in a seated position, with the arm resting on the top of the school bench, the back resting on the back of the chair, and the feet resting on the floor. The measurement was performed in the classroom in the presence of a teacher. Measurements were taken when the children had not been exposed to stress or exercise, nor immediately after a meal. Child-specific cuffs were used, appropriate to the circumference of the arm [35].

The systolic and diastolic arterial blood pressure readings were calculated as the arithmetic mean of two measurements [36]. In a situation where the difference between the readings of 1st and 2nd measurements was over 5 mmHg, a third measurement was made. The obtained blood pressure readings were interpreted basing on the blood pressure percentile grids for the Polish child population [10,16,17].

### 2.4. Statistics

Statistical analysis of the results was performed using Microsoft Excel (Microsoft, Intentional Software, Washington, DC, USA) and Statistica software (Stat Soft, Krakow, Poland). It was assumed that a level of statistical significance of α < 0.05, where *p* < 0.05, indicated significant differences, while values of *p* > 0.05 were interpreted as insignificant. Descriptive statistics were computed to characterize blood pressure values by school class, gender, and the five individual measurements. For this purpose, the average values, median, and standard deviation were calculated at the level of class profile (GC, SC), gender, and age. The normal distribution of the results was examined using the Shapiro–Wilk test. Levene’s test was used to check the homogeneity of variance. Based on the results of the calculations, a *t*-test was used to compare the mean blood pressure between the class profile (GC and SC) and gender, and at the initial and the final measurement as well.

## 3. Results

### 3.1. Systolic Blood Pressure

Between the initial and final measurement sessions, the average systolic blood pressure in the whole group increased from 110.17 mmHg to 114.52 mmHg (1.95 mmHg per year). The mean increase in the GC children was 5.88 mmHg (2.44 mmHg per year), and in the SC ones, it was 1.18 mmHg (0.59 mmHg per year) (Table 1). On average, the SC children had lower systolic blood pressure than the GC ones (110.73 mmHg and 112.92 mmHg, respectively). In the GC group, the minimal and maximal systolic blood pressure were 78.00 and 140.00 mmHg, and in the SC one, 80.00 mmHg and 139.00 mmHg, respectively. In the initial measurement session, the average systolic blood pressure did not differ significantly between the SC and GC children. However, statistical analysis showed significant differences between the GC children and the SC ones in the third, fourth, and fifth measurement sessions (Table 1).

Over the entire study period, the mean systolic blood pressure was more favourable in the SC children than in the GC ones. The average from all measurements showed that normal systolic blood pressure was found in 82.73% of children (GC 81.17%; SC 84.29%; *p* = 0.0010), hypertension in 10.12% (GC 11.27%; SC 8.96%; *p* = 0.0000), and prehypertension in 7.15% (GC 7.56%; SC 6.75%; *p* = 0.0000) (Table 2).

In girls, an increase in mean systolic blood pressure from 109.56 mmHg (the initial measurement session) to 115.28 mmHg (the final one) was observed (2.86 mmHg per year) (Table 1). The SC girls had, on average, lower systolic blood pressure compared to the GC ones (Table 1). The average from all measurements showed that normal blood pressure was found in 83.46% of girls (GC 81.83%; SC 85.10%), hypertension in 9.81% (GC 11.05%; SC 8.58%), and prehypertension in 6.72% (GC 7.12% and SC 6.32%). Girls had, on average, lower systolic blood pressure than boys. However, in boys, the mean systolic blood pressure increase was lower than in girls (from 110.76 to 113.85 mmHg; 1.95 mmHg per year) (Table 1). Similarly to girls, the SC boys had, on average, lower systolic blood pressure compared to the GC ones. The averaged results of all measurements showed that normal systolic blood pressure was found in 81.99% of boys (GC 80.51%; SC 83.47%), hypertension in 10.43% (GC 11.49%; SC 9.36%), and prehypertension in 7.58% (GC 8%; SC 7.17%).

Generally, between the initial and the final measurement sessions, the percentage of SC children with normal systolic blood pressure increased significantly from 83.15% to 86.13% (*p* = 0.0000) (Table 2). However, in the GC group, the percentage of children with normal blood pressure decreased significantly from 83.25% to 78.03% (*p* = 0.0001). The prevalence of hypertension decreased significantly in the SC children from 9.60% to 7.75% (*p* = 0.0010) but increased significantly in the GC ones from 9.82% to 13.12% (*p* = 0.0000). The frequency of prehypertension decreased slightly in the SC children from 7.25% to 6.12% and increased significantly in the GC ones from 6.92% to 8.85% (*p* = 0.0242).

**Figure 1:**
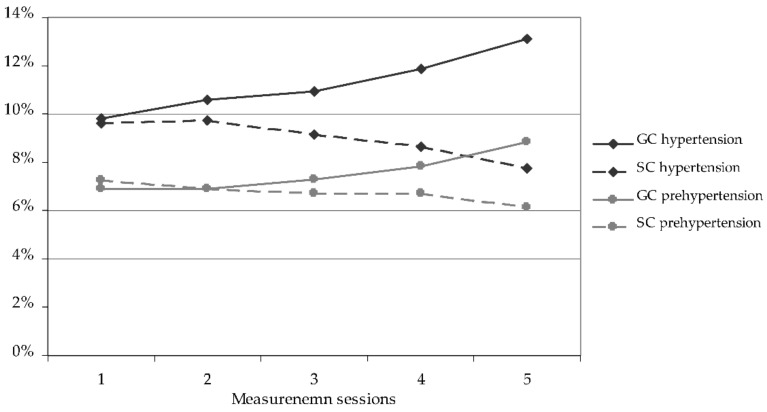
Changes in the percentage of children with systolic prehypertension and hypertension during the course of the study. GC—general education classes (standard PE, 4 h a week); SC—sport classes (elevated PE, 10 h a week).

Comparison between the initial and the final measurement sessions indicated favourable changes in systolic blood pressure in the SC children and unfavourable changes in the GC ones (Figure 1). In the SC group, the percentage of children with normal systolic blood pressure significantly increased, systolic hypertension significantly decreased, and systolic prehypertension decreased insignificantly. Conversely, in the GC group, the percentage of children with normal blood pressure significantly decreased, hypertension increased significantly, and prehypertension increased significantly as well.

**Figure 2:**
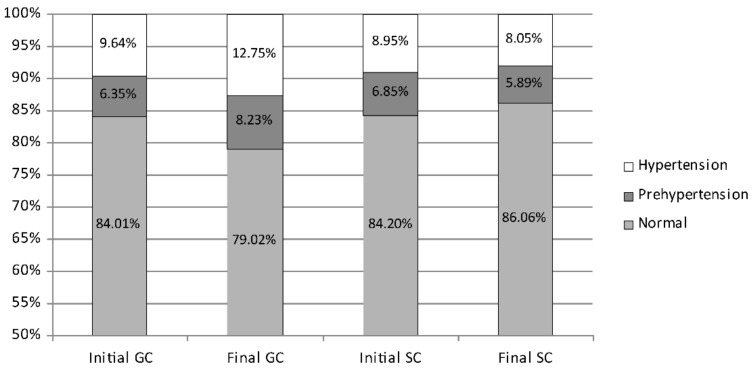
The percentage of girls with systolic prehypertension, hypertension, and normal blood pressure—comparison between the initial and the final measurement sessions. GC—general education classes (standard PE, 4 h a week); SC—sport classes (elevated PE, 10 h a week).

After 2 years, the average systolic blood pressure in girls, starting from a similar level in both groups, showed favourable changes in the SC girls and unfavourable in the GC ones (Figure 2). In the initial measurement session, normal systolic blood pressure was found in 84% of girls (GC 84.01%; SC 84.20%), prehypertension in 6.6% (GC 6.35%; SC 6.85%), and hypertension in 9% (GC 9.64%; SC 8.95%). During the course of the study, the percentage of girls with normal systolic blood pressure significantly decreased in the GC group from 84.01% to 79.02% (*p* = 0.0001) but slightly increased in the SC group from 84.20% to 86.06% (*p* = 0.0650). The percentage of girls with elevated blood pressure increased significantly in the GC group from 16.00% to 21.00% (prehypertension from 6.35% to 8.25%; hypertension from 9.65% to 12.75%; *p* = 0.0000 and *p* = 0.0001, respectively). However, it decreased in the SC group from 15.80% to 13.94% (prehypertension from 6.85% to 5.89%; hypertension from 8.95% to 8.05%; *p* = 0.0001 and *p* = 0.0000, respectively).

**Figure 3:**
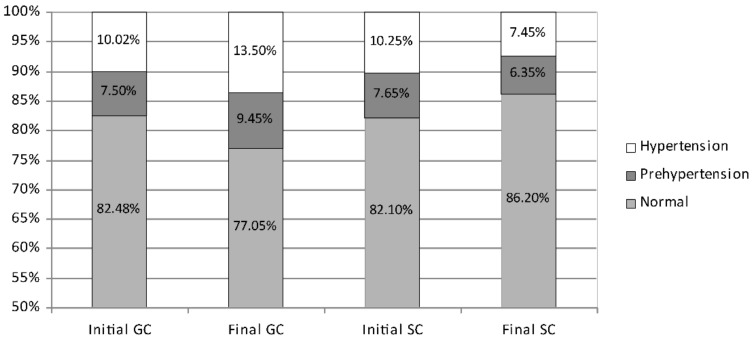
The percentage of boys with systolic prehypertension, hypertension, and normal blood pressure—comparison between the initial and the final measurement sessions. GC—general education classes (standard PE, 4 h a week); SC—sport classes (elevated PE, 10 h a week).

While the average systolic blood pressure in boys started at a similar level in both groups, after 2 years, it showed favourable changes in the SC boys and unfavourable changes in the GC boys (Figure 3). In the initial measurement session, normal systolic blood pressure was noted in 82% of boys (GC 82.48%; SC 82.10%), prehypertension in 7.57% (GC 7.50%; SC 7.65%), and hypertension in 10.13% (GC 10.02%; SC 10.25%). During the course of the study, the percentage of boys with normal systolic blood pressure in the GC group decreased significantly from 82.48% to 77.05% (*p* = 0.0001) but increased significantly in the SC group from 82.10% to 86.20% (*p* = 0.0000). The percentage of boys with elevated systolic blood pressure increased significantly in the GC group from 17.52% to 22.95% (prehypertension from 7.50% to 9.45%; hypertension from 10.02% to 13.50%; *p* = 0.0000 and *p* = 0.0023, respectively). Conversely, in the SC group, the percentage decreased from 18.00% to 13.80% (slightly in prehypertension from 7.65% to 6.35%; significantly in hypertension from 10.25% to 7.45%; *p* = 0.0502 and *p* = 0.0020, respectively).

### 3.2. Diastolic Blood Pressure

Between the initial and the final measurement sessions, a small increase in the mean diastolic blood pressure was noted (from 69.26 mmHg to 70.14 mmHg) (Table 3). In the GC group, the mean increase in diastolic blood pressure was 1.57 mmHg (0.78 mmHg per year); in the SC one, it was 0.2 mmHg (0.1 mmHg per year). The GC children had on average higher diastolic blood pressure than the SC ones (GC 69.87 mmHg; SC 68.13 mmHg). In the first and second measurement sessions at the beginning of the study, the mean diastolic blood pressure in the GC and the SC groups were similar (*p* = 0.2992 and *p* = 0.2143, respectively), while in the third, fourth, and fifth measurement session, significant differences occurred (*p* = 0.0108, *p* = 0.0311 and *p* = 0.0079, respectively).

The average from all measurements showed that normal diastolic blood pressure was found in 84.82% of children (GC 83.15%; SC 86.48%; *p* = 0.0010), diastolic hypertension in 8.45% (GC 9.68%; SC 7.22%; *p* = 0.0000), and prehypertension in 6.73% (GC 7.17%; SC 6.30%; *p* = 0.0002) (Table 4).

In girls, between the initial and the final measurement sessions, the average diastolic blood pressure slightly increased from 68.59 mmHg to 70.51 mmHg (0.96 mmHg per year). In the initial measurement session, the average diastolic blood pressure was similar in the GC girls and the SC ones (*p* = 0.6758). However, after 2 years, the diastolic blood pressure was found to be significantly higher in the GC girls than in the SC ones (*p* = 0.0042). During the course of the study, normal diastolic blood pressure was more frequently noted in the SC girls than the GC ones (GC 84.13%; SC 87.92%; *p* = 0.0000). Hypertension was more frequent in the GC girls than the SC ones (GC 9.29%; SC 6.79%; *p* = 0.0001), and similarly, prehypertension was more frequent in the GC girls than the SC ones (GC 6.58%; SC 5.30%; *p* = 0.0011). However, in boys, between the initial and the final measurement sessions, there was a slight decrease in mean diastolic blood pressure (from 69.92 mmHg to 69.78 mmHg; *p* = 0.0500). On average, in the final measurement session, the GC boys had a diastolic blood pressure slightly higher than the SC ones (GC 70.30 mmHg; SC 69.26 mmHg; *p* = 0.3777). Averaged from all measurement sessions, normal diastolic blood pressure was found in 83.62% of boys and was slightly more common in the SC group than in the GC one (GC 82.21%; SC 85.03%; *p* = 0.0001). Diastolic hypertension was more often found in the GC than the SC boys (GC 10.07%; SC 7.63%; *p* = 0.0000). The percentage of boys with diastolic prehypertension was similar in both groups (GC 7.72%; SC 7.34%; *p* = 0.0550).

During the course of the study, the percentage of SC children (boys and girls) with normal diastolic blood pressure gradually increased (from 84.94% to 88.50%; *p* = 0.0001), diastolic hypertension slightly decreased (from 8.14% to 6.25%; *p* = 0.0011), and diastolic prehypertension significantly decreased (from 6.92% to 5.25%; *p* = 0.0000) (Table 4). Conversely, in the GC group, the percentage of children with normal diastolic blood pressure significantly decreased (from 85.70% to 80.20%; *p* = 0.0001), diastolic hypertension significantly increased (from 7.75% to 11.77%; *p* = 0.0022), and diastolic prehypertension slightly increased (from 6.55% to 8.03%; *p* = 0.0011).

**Figure 4:**
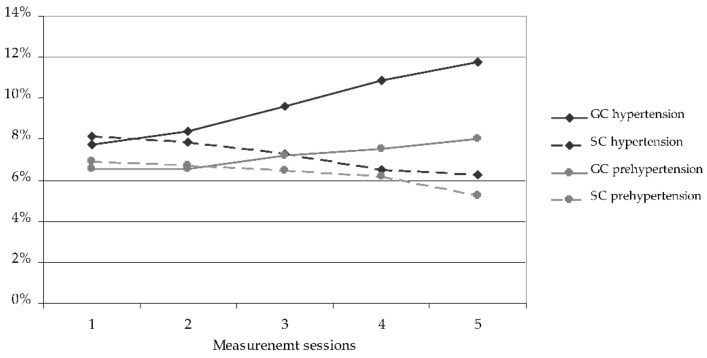
Changes of the percentage of children with diastolic prehypertension and hypertension during the course of the study. GC—general education classes (standard PE, 4 h a week); SC—sport classes (elevated PE, 10 h a week).

Comparison between the initial and the final measurement sessions showed favourable changes in diastolic blood pressure in the SC children and unfavourable changes in the GC ones. In the SC group, the percentage of children with normal diastolic blood pressure increased, hypertension decreased, and prehypertension decreased (Figure 4). However, in contrast, in the GC group, the percentage of children with normal blood pressure significantly decreased, hypertension significantly increased, and prehypertension increased insignificantly (Figure 4).

**Figure 5:**
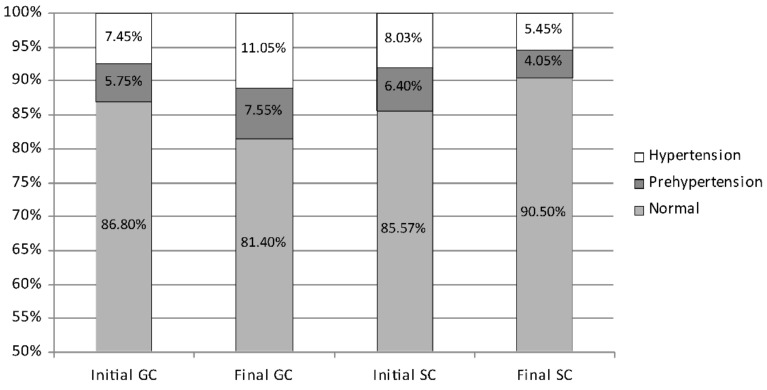
The percentage of girls with diastolic prehypertension, hypertension, and normal blood pressure—comparison between the initial and the final measurement sessions. GC—general education classes (standard PE, 4 h a week); SC—sport classes (elevated PE, 10 h a week).

While the average diastolic blood pressure in girls started at a similar level in both groups, after 2 years, it showed favourable changes in the SC group and unfavourable changes in the GC one (Figure 5). In the initial measurement session, normal diastolic blood pressure was shown in 86.10% of girls (GC 86.80%; SC 85.57%), prehypertension in 6.07% (GC 5.75%; SC 6.40%), and hypertension in 7.74% (GC 7.45%; SC 8.03%). During the course of the study, the percentage of girls with normal diastolic blood pressure decreased significantly in the GC group from 86.80% to 81.40% (*p* = 0.0002) but increased significantly in the SC one from 85.57% to 90.50% (*p* = 0.0001). The percentage of girls with elevated diastolic blood pressure increased significantly in the GC group from 13.20% to 17.60% (prehypertension from 5.75% to 7.55% and hypertension from 7.45% to 11.05%; *p* = 0.0004 and *p* = 0.0002, respectively). However, the percentage decreased significantly in the SC group from 14.43% to 9.50% (prehypertension from 6.40% to 4.05% and hypertension from 8.03% do 5.45%; *p* = 0.0001 and *p* = 0.0003, respectively).

**Figure 6:**
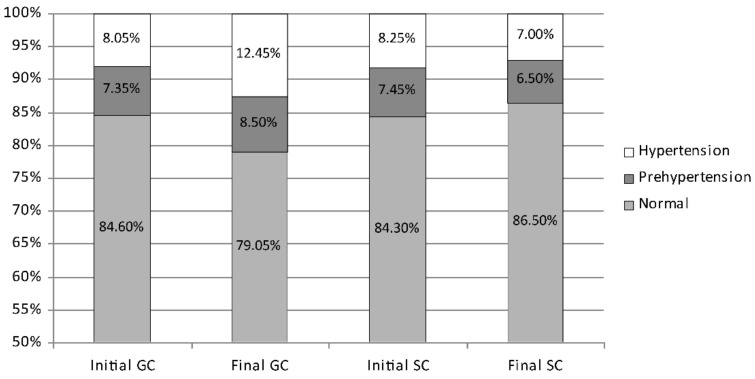
The percentage of boys with diastolic prehypertension, hypertension and normal blood pressure—comparison between the initial and the final measurement sessions. GC—general education classes (standard PE, 4 h a week); SC—sport classes (elevated PE, 10 h a week).

In the initial measurement session, the boys’ average diastolic blood pressure was similar in both the GC group and the SC one. However, after 2 years, favourable changes in the SC boys and unfavourable in the GC ones were found (Figure 6). In the initial measurement session, normal diastolic blood pressure was shown in 84.45% of boys (GC 84.60%; SC 84.30%), prehypertension in 7.40% (GC 7.35%; SC 7.45%), and hypertension in 8.15% (GC 8.05%; SC 8.25%). During the course of the study, the percentage of boys with normal diastolic blood pressure decreased significantly in the GC group from 84.60% to 79.05% (*p* = 0.0000) but increased significantly in the SC one from 84.30% to 86.50% (*p* = 0.0002). The percentage of boys with elevated diastolic blood pressure increased significantly in the GC group from 15.40% to 20.95% (prehypertension from 7.35% to 8.50% and hypertension from 8.05% to 12.45%; *p* = 0.0000 and *p* = 0.0003, respectively). However, it decreased slightly in the SC group from 15.70% to 13.50% (prehypertension from 7.45% to 6.50% and hypertension from 8.25% to 7.00%).

Summarizing these results, a favourable impact of increased PA at school, by elevation of the number of compulsory PE lessons to 10 h a week, was shown. Starting from a similar level, during the study, the number of children with normal blood pressure decreased in the standard PE group (GC) from 83.25% to 78.03% but increased in the elevated PE one (SC) from 83.15% to 86.13%. The prevalence of elevated blood pressure (both prehypertension and hypertension) increased by one-third in the standard PE group (GC) from 16.74% to 21.97% but decreased by more than one-sixth in the elevated PE one (GC) from 16.85% to 13.87%. The prevalence of hypertension itself also increased by one-third in the standard PE children from 9.82% to 13.12% but decreased in elevated PE ones by one-fifth from 9.60% to 7.75%. The trends of the changes were similar for both girls and boys.

## 4. Discussion

Many studies have shown a beneficial effect of increasing PA in reducing the risk of hypertension in children and adolescents [29,30,37]. Orlando et al. (2018), like many others, showed that hypertension and prehypertension were positively correlated with a low PA level in children [31,32,33,37]. In this study, it was found that increasing the number of compulsory PE lessons in the school timetable resulted in a decrease in the percentage of children with hypertension and prehypertension. However, in some studies, the relationship between PA and blood pressure in children was not confirmed [26,38].

Generally, the prevalence of hypertension in children and adolescents has been increasing in recent years. This is explained by increasing sedentary behaviour and low PA, as well as the unhealthy diet of many children [39,40]. It is estimated that hypertension affects more than 10% of school-age children [41,42,43]. Our study found a similar level; however, it was lower in the children with an elevated number of PE lessons. Moreover, after 2 years of follow-up, at the age of approx. 12 years on average, nearly 17% of children had elevated blood pressure (both hypertension and prehypertension). However, it was related to the PA and concerned nearly 22% of the low-PA children (4 h of PE a week) and nearly 14% of the elevated-PA ones (10 h).

The prevalence of high blood pressure in children differs by region [41,42,44]. Studies in European countries have shown that there is a large difference in the prevalence of high blood pressure in children from central Europe compared to southern Europe [45]. It was shown that elevated blood pressure occurred in an average 16.4% of children and adolescents from central Europe [45]. However, studies from Greece showed an alarming percentage of children with hypertension, reaching almost 23% [46]. Other reports also indicated a significant increase in the percentage of children with hypertension in the last decade [3,47]. Looking back, Kułaga et al. (2011), in a study from over 12 years ago, showed that the prevalence of hypertension in children and adolescents in Poland was much lower than today and reached only 3.5% [48]. However, in the current study, an average of 10% of children had hypertension, i.e., almost three times higher. Many studies have indicated that the risk of hypertension in children increases with age and is slightly higher in boys than in girls [39,49,50]. This was confirmed in this study, but only in the case of standard-PE children. In the case of elevated PE, the number of hypertensive children decreased during the study period. Generally, it is estimated that the prevalence of elevated blood pressure (prehypertension and hypertension) among school-age children ranges from 12% to 17% [51,52,53]. Moreover, it is well established that children with elevated blood pressure are between two and three times more likely to develop hypertension in adulthood [54].

Normal systolic and diastolic blood pressure was noted in an average of 80% of children aged 10 to 12 years of the standard PE group; however, the percentage decreased with age. In a study by Bell et al. (2019) involving children and adolescents aged 10–17 years, 71% of participants had normal blood pressure, which also decreased with age [47]. However, in a study by Hardy et al. (2021), systolic blood pressure in children aged 8–12 years was relatively stable over the years of observation [55]. Our research, however, showed that blood pressure in children varied significantly depending on the level of PA at school, corresponding to the curricular number of PE lessons. Similarly, García-Hermoso et al. (2020) showed that an additional form of PA in children aged 8–10 years had a favourable effect on the reduction in blood pressure [56].

Older studies, such as a study by Kelley et al. (2003), indicated that PA had little effect on lowering blood pressure in children [57]. That was probably due to a lower incidence in the past of elevated blood pressure in children. More recent studies such as the study by Grace et al. (2021) have shown that a ten-week intervention of elevated PA and nutritional education in overweight and obese adolescents had a beneficial effect on lowering blood pressure [58]. Similarly, Wellman et al. (2020) and Carson et al. (2014) showed that PA had a strong effect on reducing the prevalence of hypertension [59,60]. Numerous studies have shown that elevated blood pressure in childhood increases the risk of hypertension as adults [13,16,17,39]. The results obtained in this study indicated that early prevention of hypertension in children can be successfully supported by increased PA at school by an increase in the number of compulsory PE lessons.

Despite the demonstration of significant trends in children’s blood pressure, the study presented herein has certain limitations. The study covered only 2 years of observation. It was planned to continue the research on the same group of children until they graduated from primary school (approx. age 15 years). However, the outbreak of the COVID-19 pandemic and the associated frequent lockdowns and implementation of online education resulted in discontinuation of the study. Moreover, the study was limited to only a relatively small sample of children between the ages of 10 and 12 from Siedlce. It would be worthwhile to extend the research to children in other age groups and from other areas. Moreover, the only measured variable influencing blood pressure was the number of curricular PE lessons (4 and 10 h a week). Extracurricular activity should also be measured in order to summarize the overall PA level of children participating in the study. Another limitation is that blood pressure is influenced not only by PA but also by nutritional behaviour; however, this analysis did not take this into account. It is advisable to extend the research by including the assessment of nutritional behaviour and extracurricular PA of children to more widely demonstrate the effect of elevated PA on children’s blood pressure.

## 5. Conclusions

The majority of children aged 10 to 12 years showed normal blood pressure numbers during the two-year follow-up. However, increasing the number of PE lessons in the school timetable (from 4 to 10 h a week) favourably impacted the children’s blood pressure over time. Starting from a similar level, during the 2 years of the study, the number of children with normal blood pressure decreased in children with a standard number of PE lessons but increased in the elevated-PE group. Consequently, the number of children with elevated blood pressure increased in children with a standard number of PE lessons but decreased in the elevated-PE group. Trends in the changes were similar for both girls and boys. Increased PA at school by increasing the number of PE lessons benefits children’s arterial blood pressure over time. It can be concluded that early prevention of hypertension in children can be supported by an adequate number of PE lessons. It is recommended to increase the number of PE lessons in the school timetable, which brings measurable health benefits.

## Figures and Tables

**Table 1:** The mean systolic blood pressure in children during the course of the study (mmHg).

Measurement Session	Average Age (Years)	Systolic Blood Pressure (mmHg)											
		Mean Total	GC					SC					*p*
			Mean	Median	Min.	Max.	SD	Mean	Median	Min.	Max.	SD	
Average													
I	10.27	110.17	110.25	109.00	84.00	140.00	12.67	110.09	110.00	84.00	139.00	10.43	0.9176
II	10.90	108.52	109.52	109.00	79.00	138.00	10.59	107.53	105.50	80.00	136.00	10.61	0.1421
III	11.27	113.00	114.12	114.00	86.00	137.00	12.19	111.87	112.00	85.00	133.00	10.06	**0.0117**
IV	11.90	112.92	114.58	114.00	78.00	135.00	12.16	111.27	115.00	86.00	130.00	13.84	**0.0208**
V	12.26	114.52	116.13	115.50	87.00	131.00	12.93	112.92	112.00	89.00	130.00	9.96	**0.0208**
Girls													
I	10.27	109.56	110.03	109.00	84.00	133.00	12.49	109.09	110.00	84.00	131.00	10.11	0.6543
II	10.90	107.91	108.73	109.00	79.00	132.00	11.30	107.09	107.00	80.00	130.00	11.04	0.4275
III	11.27	113.88	114.96	115.00	89.00	132.00	12.68	112.81	113.00	79.00	129.00	11.24	0.3323
IV	11.90	112.29	113.12	113.00	78.00	128.00	11.74	111.47	113.00	86.00	126.00	10.14	0.4156
V	12.26	115.28	117.42	116.00	87.00	130.00	13.60	113.14	114.00	89.00	127.00	9.19	**0.0466**
Boys													
I	10.27	110.76	110.43	108.50	87.00	140.00	12.92	111.09	110.00	91.00	139.00	10.74	0.7563
II	10.90	109.08	110.21	109.50	85.00	138.00	9.69	107.96	105.00	81.00	136.00	10.25	0.2133
III	11.27	112.16	113.39	113.50	86.00	137.00	11.79	110.93	111.00	85.00	133.00	8.72	0.1870
IV	11.90	113.46	115.84	116.00	92.00	135.00	12.47	111.08	111.08	87.00	130.00	9.85	**0.0189**
V	12.26	113.85	115.01	114.00	96.00	131.00	12.32	112.70	113.00	94.00	130.00	8.38	0.2227

GC—general education classes (standard PE, 4 h a week); SC—sport classes (elevated PE, 10 h a week); SD—standard deviation; measurement sessions: I—September 2017; II—March 2018; III—September 2018; IV—March 2019; V—September 2019. Bold font reflects significant difference.

**Table 2:** The changes in the percentage of children in particular systolic blood pressure categories from the initial to the final measurement sessions (%).

Variables	Measurement Session									
	I		II		III		IV		V	
Average age (years)	10.27		10.90		11.27		11.90		12.26	
Class type	GC	SC	GC	SC	GC	SC	GC	SC	GC	SC
Normal	83.25	83.15	82.52	83.36	81.75	84.15	80.30	84.66	78.03	86.13
Prehypertension	6.92	7.25	6.92	6.92	7.30	6.70	7.83	6.70	8.85	6.12
Hypertension	9.82	9.60	10.57	9.72	10.95	9.15	11.87	8.64	13.12	7.75

GC—general education classes (standard PE, 4 h a week); SC—sport classes (elevated PE, 10 h a week); measurement sessions: I—September 2017; II—March 2018; III—September 2018; IV—March 2019; V—September 2019.

**Table 3:** The mean diastolic blood pressure in children during the course of the study (mmHg).

Measurement Session	Average Age (Years)	Diastolic Blood Pressure (mmHg)											
		Mean Total	GC					SC					*p*
			Mean	Median	Min.	Max.	SD	Mean	Median	Min.	Max.	SD	
Average													
I	10.27	69.26	69.79	70.00	52.00	90.00	7.95	68.73	67.00	52.00	89.00	8.93	0.2992
II	10.90	67.90	68.40	69.00	54.00	89.00	7.53	67.39	67.00	53.00	89.00	5.91	0.2143
III	11.27	68.70	70.00	69.00	56.00	90.00	8.95	67.40	67.00	52.00	85.00	7.06	**0.0108**
IV	11.90	69.02	69.80	69.00	53.00	89.00	7.80	68.24	68.00	53.00	87.00	7.85	**0.0311**
V	12.26	70.14	71.36	70.00	55.00	92.00	9.67	68.93	69.00	51.00	86.00	9.97	**0.0079**
Girls													
I	10.27	68.59	68.92	70.00	52.00	86.00	8.87	68.27	67.00	52.00	82.00	7.99	0.6758
II	10.90	67.12	67.43	69.00	54.00	85.00	6.94	66.81	67.00	53.00	79.00	4.93	0.5757
III	11.27	68.40	69.26	69.00	56.00	81.00	7.79	67.54	67.00	52.00	80.00	8.37	0.2506
IV	11.90	68.54	69.35	69.00	53.00	85.00	5.84	67.73	67.00	53.00	87.00	6.06	0.1445
V	12.26	70.51	72.42	72.00	55.00	86.00	7.95	68.60	69.00	51.00	84.00	6.19	**0.0042**
Boys													
I	10.27	69.92	70.66	70.00	56.00	90.00	7.03	69.19	68.00	53.00	89.00	9.82	0.3315
II	10.90	68.66	69.36	69.00	57.00	89.00	7.95	67.96	68.00	56.00	89.00	6.75	0.2902
III	11.27	68.99	70.72	68.00	59.00	90.00	9.85	67.26	67.00	54.00	85.00	5.52	**0.0171**
IV	11.90	69.49	70.24	70.00	56.00	89.00	6.28	68.75	69.00	59.00	87.00	4.45	0.1290
V	12.26	69.78	70.30	69.00	66.00	92.00	7.58	69.26	70.00	57.00	86.00	5.37	0.3777

GC—general education classes (standard PE, 4 h a week); SC—sport classes (elevated PE, 10 h a week); SD—standard deviation; measurement sessions: I—September 2017; II—March 2018; III—September 2018; IV—March 2019; V—September 2019. Bold font reflects significant difference.

**Table 4:** The changes in the percentage of children in particular diastolic blood pressure categories from the initial to the final measurement sessions (%).

Variables	Measurement Session									
	I		II		III		IV		V	
Average age (years)	10.27		10.90		11.27		11.90		12.26	
Class type	GC	SC	GC	SC	GC	SC	GC	SC	GC	SC
Normal	85.70	84.94	85.05	85.42	83.18	86.23	81.60	87.30	80.20	88.50
Prehypertension	6.55	6.92	6.55	6.73	7.20	6.47	7.53	6.17	8.03	5.25
Hypertension	7.75	8.14	8.37	7.85	9.62	7.30	10.87	6.53	11.77	6.25

GC—general education classes (standard PE, 4 h a week); SC—sport classes (elevated PE, 10 h a week); measurement sessions: I—September 2017; II—March 2018; III—September 2018; IV—March 2019; V—September 2019.

## Data Availability

Data are available upon request.
